# Successful management of primary sarcoma of the breast with complete pathological response: a case report

**DOI:** 10.1093/jscr/rjad677

**Published:** 2023-12-18

**Authors:** Hasan Arafat, Ola Abulaban-Awar, Mohammad Fatayer, Marwan Abufara

**Affiliations:** Department of Internal Medicine, Augusta Victoria Hospital, P.O. 19178, Rabia Al-Adawiyya Street, East Jerusalem, 91191, Palestine; Cancer Care Center, Augusta Victoria Hospital, P.O. 19178, Rabia Al-Adawiyya Street, East Jerusalem, 91191, Palestine; Department of Pathology, Augusta Victoria Hospital, P.O. 19178, Rabia Al-Adawiyya Street, East Jerusalem, 91191, Palestine; Surgical Care Center, Augusta Victora Hospital, P.O. 19178, Rabia Al-Adawiyya Street, East Jerusalem, 91191, Palestine

**Keywords:** sarcoma, breast cancer, radiotherapy, chemotherapy, rare neoplasm

## Abstract

Primary sarcomas of the breast are extremely rare and heterogenous malignancies; they should be differentiated from phyllodes tumors. They are characterized by their high rate of recurrence, rapid growth and aggressive coarse. We present a case of a 41-year-old Palestinian female who presented with a recurrent breast mass. Biopsy showed primary sarcoma and imaging confirmed metastasis to axillary lymph nodes. The patient received six cycles of ifosfamide–adriamycin protocol resulting in complete pathological response. She underwent left sided modified radical mastectomy followed by radiation and six more cycles of the same protocol as adjuvant. Primary breast sarcomas are rare neoplasms that require multidisciplinary discussion to guide treatment. The approach to these tumors is chemotherapy followed by surgical resection when operable, in addition to local control via radiotherapy and adjuvant chemotherapy.

## Introduction

Primary breast sarcomas are rare soft tissue tumors. They can be either primary (de novo) or secondary. Their presentation masquerades that of breast carcinomas [1]. Most cases of breast sarcoma are seen secondary to radiotherapy following breast conservative treatment. Their presentation is characterized by rapid growth when compared to epithelial carcinomas. Histopathological analysis is cornerstone for exclusion of phyllodes tumors and metaplastic breast carcinomas [2]. We present a rare case of a 41-year-old female patient presenting with a recurrent breast mass, diagnosed as having primary high-grade breast sarcoma with axillary lymph node metastasis, demonstrating complete pathological response following neoadjuvant chemotherapy.

## Case report

A 41-year-old Palestinian single female patient, with medical history of diabetes mellitus type two and a benign fibroadenoma excised in 2014, presented to general surgery clinic due to recurrence of the breast mass in May 2022, she underwent surgical excision without tissue biopsy, however, the mass recurred in August 2022. Core-tissue biopsy showed a high-grade spindle cell sarcoma.

**Figure 1:**
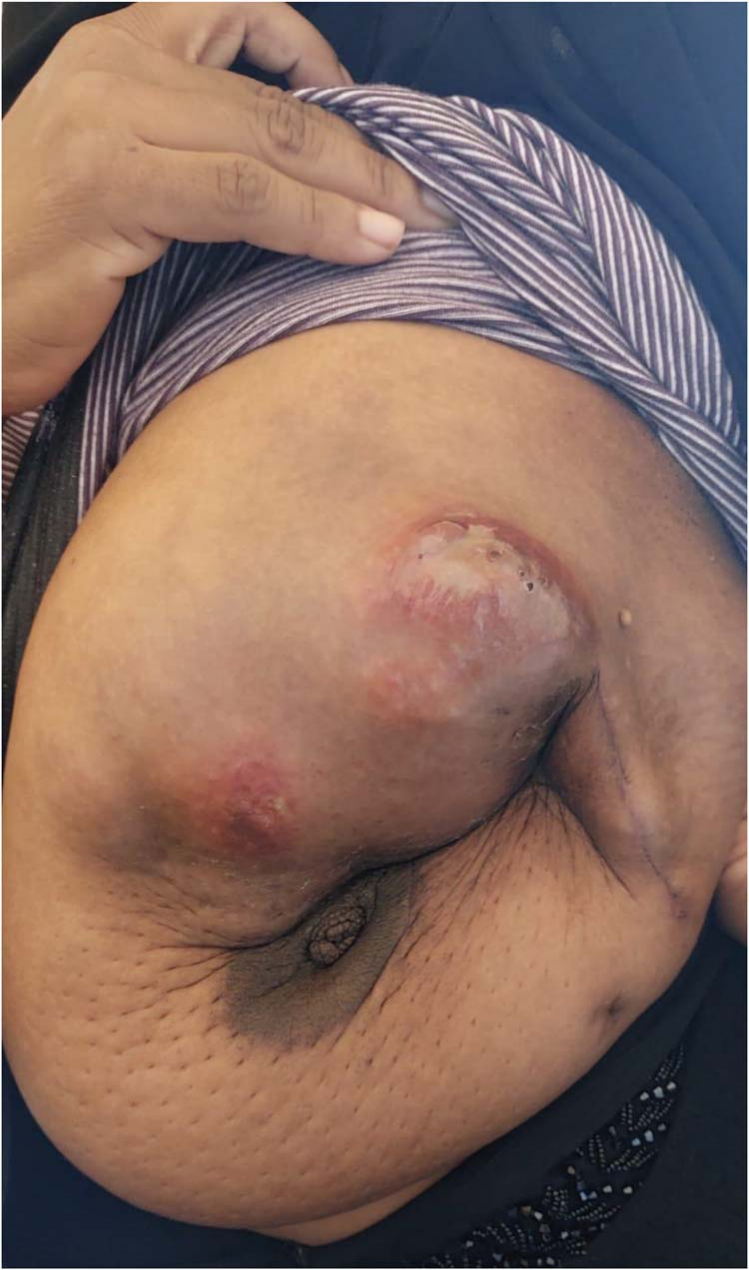
Breast lesion prior to commencing treatment.

**Figure 2:**
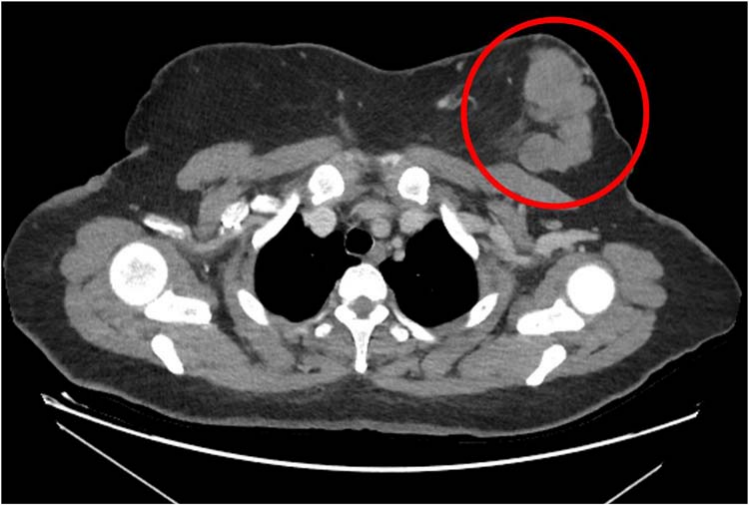
Breast mass seen on baseline CT scan, encircled.

**Figure 3:**
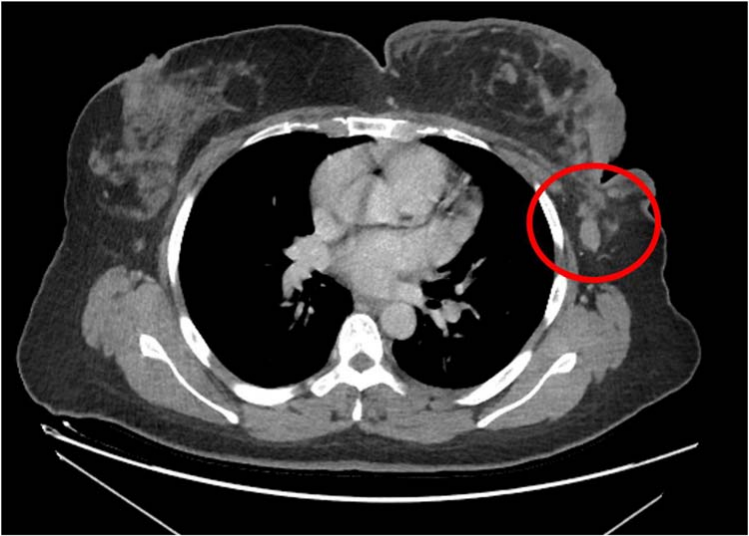
Left breast mass along with involved axillary lymph nodes, encircled.

The patient was referred to medical oncology clinic. On exam, she had an eastern cooperative oncology group-performance status of zero, was in pain from her left breast. Assessment of the breast lump revealed a palpable, huge left breast mass, hard in consistency, around 10 cm in largest diameter. There were no palpable lymph nodes in the left axilla. Examination of the right breast was unremarkable. The affected breast is shown in Fig. 1. Otherwise, her chest and abdomen exam were unremarkable, with no lower limb edema. Baseline chest-abdomen–pelvis computed tomography (CT), shown in Fig. 2, showed a large left breast mass 8.0× 2.0 × 4.7 cm with diffuse skin thickening, few axillary lymph adenopathy, the largest of which measured 1.9 cm (shown in Fig. 3), with no distant metastasis. No magnetic resonance imaging was obtained prior to treatment as it was unavailable at the institute. The case was discussed by a multidisciplinary team of medical oncologists, radiation oncologists, surgical oncologists and pathologists. The final decision was that the patient should be treated as a case of high-grade sarcoma, so she was started on ifosfamide–adriamycin (IA) protocol (ifosfamide, 2500 mg/m^2^_,_ mesna, 800 mg/m^2^ and doxorubicine, 75 mg/m^2^ intravenous (IV) for 3 days) as neoadjuvant, to be followed by surgery and adjuvant radiotherapy and chemotherapy. Paraffin blocks were requested by the pathologists for case revision at our institute.

Due to the severity of her pain, the patient was evaluated by the pain team during her first admission, she was started on morphine sulfate tablets, 30 mg twice daily per os (PO), morphine 5 mg IV six times per day as needed, in addition to pregabalin, 75 mg PO twice daily. She reported remarkable improvement during her three days stay in hospital. IV morphine was stopped and she was prescribed morphine immediate release tablets, 15 mg PO four times per day as needed and discharged home.

**Figure 4:**
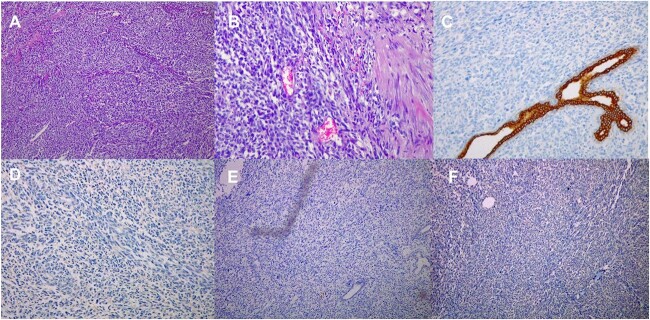
Histological images (10 × magnification) of the formalin-fixed paraffin-embedded sections (A) shows malignant spindle cell tumor with prominent cytological atypia. (B) shows malignant spindle cell tumor with prominent cytological atypia and pleomorphism. (C) Immunohistochmeically-stained slide of Ckpan showing negative reaction of tumor cells. (D) Immunohistochmeically-stained slide of ER showing negative reaction of tumor cells. (E) Immunohistochmeically-stained slide of desmin and myogenin showing negative reaction of tumor cells. (F) Immunohistochmeically-stained slide of CD56 showing negative reaction of tumor cells.

Paraffin blocks were brought for case revision as per hospital policy. Histopathology examination (Fig. 4) showed features keeping with high-grade sarcoma, with sections showing spindle cell tumor and prominent cytological atypia. Small foci of entrapped benign epithelial breast tissue were seen, wide areas of necrosis were present, and the mitotic index was about 30/10 per high-power field. Immunohistochemistry stains showed neoplastic cells positive for vimentin and focally positive for epithelial membrane antigen (EMA), negative for pan-cytokeratin (CK), CK7, estrogen receptors (ER), CD56, calretinin, c-KIT, and B-catenin. Ki67 positive in about 80% of tumor cells. The possibility of malignant phyllodes tumor could not be ruled out.

**Figure 5:**
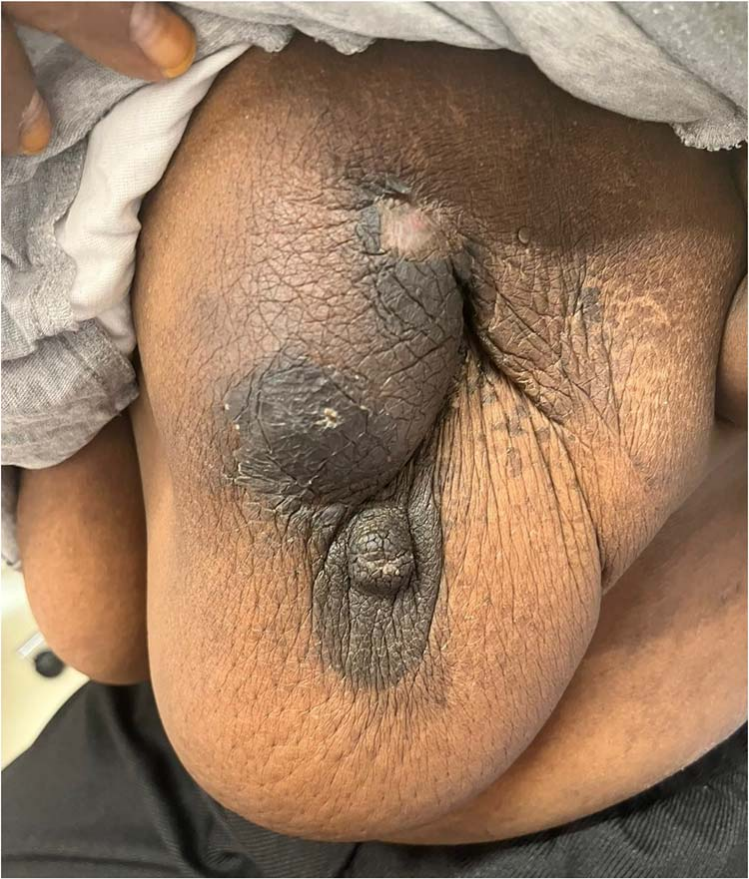
Left breast after six cycles of IA.

The patient received a total of six cycles of IA protocol, one cycle of goserelin as a means of fertility preservation, with remarkable clinical improvement, she stopped her morphine tablets (both long-duration and as needed) after the third cycle as her pain resolved, kept only on pregabalin. The mass shrunk remarkably as shown in Fig. 5.

**Figure 6:**
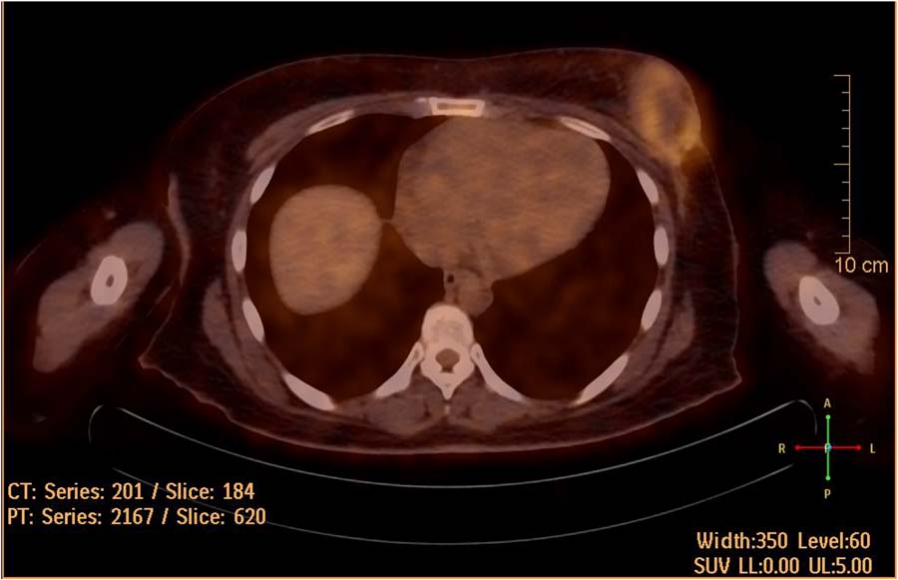
Hypermetabolic left breast mass in last positron emission tomography/computed tomography, following six cycles of IA.

She underwent positron emission tomography/computed tomography after the sixth cycle, shown in Fig. 6. It showed a left breast hypermetabolic mass, compatible with known primary disease, there was no previous study for comparison, however, comparison with previous CT showed marked interval morphological regression of the tumor, no hypermetabolic lymph nodes, with no distant metastasis. Follow up breast ultrasound showed multifocal multicentric left breast lesions with few suspicious left axillary lymph nodes. The patient was seen in the surgical oncology outpatient clinic and prepared for mastectomy.

**Figure 7:**
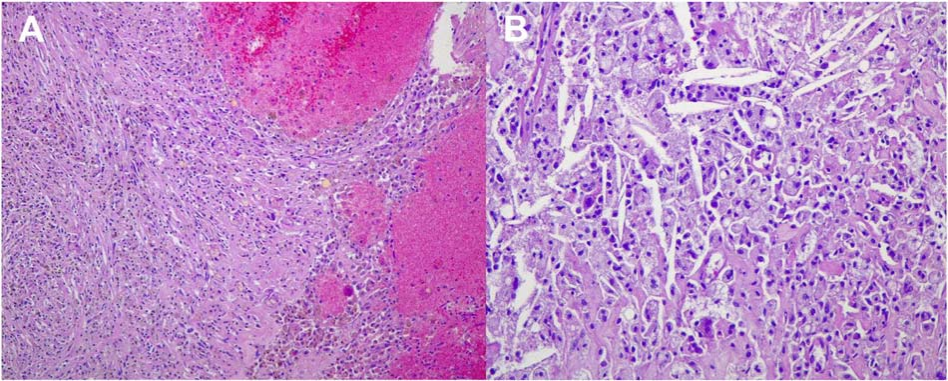
Histological images (20 × magnification) of the formalin-fixed paraffin-embedded sections. (A) shows no malignant cells and prominent interstitial hemorrhage with hemosiderin deposits due to treatment effect. (B) shows no malignant cells and numerous cholesterol clefts surrounded by foamy macrophages due to treatment effect.

On March 2023, the patient underwent left sided modified radical mastectomy with axillary lymph node dissection. Through an elliptical incision, the upper and lower flaps were dissected up to the upper and lower limits of breast tissue, the breast and pectoral fascia were excised. The axillary contents were removed after identifying the axillary vein, thoracodorsal and long thoracic nerves. The intercosto-brachial nerve was preserved. Homeostasis was secured. Red-i-vac drains were inserted. The skin was closed in -Y- shape duo to wide skin excision. The excised tissues were sent for histopathology. The patient was discharged in a good condition. Histopathology of excised tissue (shown in Fig. 7) showed fat necrosis, hemosiderin deposits, and giant cell reaction in keeping with treatment effect, but no evidence of residual malignancy. Sixteen lymph nodes were found, all free from malignancy, four of them showed fibrosis and hemosiderin deposits in keeping with treatment effect, marking complete pathological response.

In radiation oncology clinic, she was scheduled for 30 fractions of radiotherapy, with a daily dose of 2 Gy to the left breast and axilla via arc-modulated radiation therapy technique, afterwards, she received six more cycles of IA with dexrazoxane, 500 mg/m^2^ IV with each anthracycline dose as a means of cardioprotection.

## Discussion

Breast sarcomas are a rare entity, comprising <1% of breast malignancies [3]. They present as gradually, progressively enlarging mass lesion that can be associated with skin changes and pain. A presentation typical for that of sarcomas in trunk and extremities [4]. Breast sarcomas can be either primary or secondary. De novo breast sarcomas have no specific risk factors. Several genetic syndromes have been implicated in the pathogenesis of this entity, including Li–Fraumeni syndrome, type one neurofibromatosis, and familial polyposis. The most important factor for the development of secondary breast sarcoma is chest irradiation in addition to chronic lymphedema [5]. Primary breast sarcomas are divided into three classes: malignant phyllodes tumors, sarcomas arising post-irradiation, and primary breast sarcomas. The most common presentation is a palpable mass within the breast tissue. These masses are usually evaluated by a multidisciplinary team of clinicians, radiologists and pathologists [6]. Histopathology is key in the diagnosis of these tumors, with immunohistochemistry playing a major role. Distinction with phyllodes carcinoma is important for management and prognosis. Biphasic tumor, with leaf-like architecture associated with stromal overgrowth and exaggerated intracanalicular pattern of the epithelial component recognize the former. CD34 is expressed in the stroma of most phyllodes tumors. Sarcomas are differentiated by their positivity for S100, their herringbone pattern of arrangement with tapering elongated nuclei and scant cytoplasm. These cells exhibit increased mitotic activity along with the loss of CD34. The cells are reactive to smooth muscle actin (SMA) [2]. It’s recommended that sarcomas be classified based on a simple description such as spindle cell sarcoma, pleomorphic sarcoma, myxoid sarcomas, and small round cell sarcomas. Desmin, vimentin, SMA, keratin, CD34, EMA, S-100, and leukocyte common antigen should be all analyzed for sarcomas to differentiate sarcomas with a specific line of differentiation [7]. In our case, the cells were positive for vimentin and EMA, with no markers specific for a certain subtype.

Staging of breast sarcoma is based on the American Joint Committee on Cancer, it incorporates histologic grade (G), tumor size (T), nodal status (N), and the presence of distant metastasis (M) [8]. There is no consensus regarding the appropriate treatment of breast sarcomas. Neoadjuvant chemotherapy can be used in order to shrink the tumor for better surgical results and free margins [9]. Surgery with a mastectomy or wide local excision with adequate margins showed equivalent outcomes, with the addition of adjuvant radiotherapy and chemotherapy in high-risk cases [10]. The MD Anderson series of 60 patients who received either adjuvant chemotherapy or radiotherapy was associated with improved disease-free survival, however, the whole response rates to systemic therapy was disappointing [6]. As with the protocol of choice, neoadjuvant chemotherapy with an anthracycline plus ifosfamide had shown a positive result on the overall survival and relapse-free survival of high-risk soft tissue sarcomas. Evidence regarding adjuvant chemotherapy is less clear, but a combination of doxorubicin and ifosfamide offers the best evidence. Adjuvant radiotherapy had shown to reduce mortality and locoregional recurrence [5]. Despite the diagnostic differences between malignant phyllodes tumor and primary sarcoma, the mainstay of treatment remains surgical, with two factors definitely affecting the outcome—the size of the primary tumor and the excision margins [6].

Despite its significant antitumor activity, a major concern regarding its utilization as adjuvant therapy is the risk of cardiotoxicity, which is dose-dependent especially when the accumulative dose exceeds 500 mg/m^2^ [11]. Dexrazoxane is a water-soluble positive enantiomer of the racemic mixture razoxane. It’s hydrolyzed to an active form that binds iron, preventing the generation of suprahydroxide radicals, which prevents mitochondrial damage, reducing the risk of doxorubicin-induced cardiotoxicity [12]. There seems to be a synergistic cardiotoxic effect when adriamycin is combined with mediastinal irradiation, in issue clinicians should be aware of in patients receiving adriamycin at a dose <500 mg/m^2^ body surface area [13].

## Conclusion

Here we present a rare case of primary breast sarcoma with metastasis to axillary lymph nodes. Treatment of these tumors should be planned in a multidisciplinary fashion. As our patient had a metastatic, initially inoperable disease, chemotherapy was the mainstay of treatment, as disease response or stabilization is obtained in a significant minority of cases, excision of the tumor as well as metastatic sites can be discussed by a multidisciplinary team.

## Data Availability

The data that support the findings of this study are available from the corresponding author, HA, upon reasonable request.

## Declaration

Ethical approval and consent to participate obtained.
